# Relationship between nt-probnp and extravascular lung water in ards

**DOI:** 10.1186/2197-425X-3-S1-A1006

**Published:** 2015-10-01

**Authors:** SA Komarov, MY Kirov

**Affiliations:** Northern State Medical University, Arkhangelsk, Russian Federation

## Introduction

Natriuretic peptides have become important tools for diagnosis, risk stratification and therapeutic decision making for patients with heart failure and hydrostatic pulmonary edema. However, the practical use of N-terminal pro-brain natriuretic peptide (NT-proBNP) in ARDS patients and its relationship with non-cardiogenic pulmonary edema remain controversial.

## Objectives

To evaluate the relationship between plasma concentrations of NT-proBNP, hemodynamics and extravascular lung water and to assess the possibility of using NT-proBNP to diagnose pulmonary edema in ARDS.

## Methods

Fifteen adult patients with mean age of 43 (27-50) years with diagnosed ARDS according to Berlin definition were enrolled into an observational one-center prospective pilot study. The patients received hemodynamic monitoring using transpulmonary thermodilution (PiCCO_2_, Pulsion Medical Systems, Germany) with measurement of mean arterial pressure, cardiac index (CI), central venous pressure, global end-diastolic volume index (GEDVI), systemic vascular resistance index and extravascular lung water index (EVLWI). Hemodynamic, respiratory parameters and plasma concentrations of NT-proBNP were assessed on the day of admission and on days 3 and 5 of hospitalization. Patients received goal-directed therapy using CI, GEDVI and EVLWI. The statistical analysis was performed using Mann-Whitney U-test and Spearman's correlation coefficient. The data are presented as median (25th-75th percentiles).

## Results

The causes of ARDS included severe pneumonia (33%), sepsis (33%), multiple trauma (27%) and acute poisoning (7%). On admission, 8 patients had mild, 5 - moderate and 2 - severe ARDS. The ICU mortality was 27% (4 patients). The mean plasma concentrations of NT-proBNP in ARDS patients were higher than the normal values, with a trend for decrease during hospitalization: from 4706 (334-15173) pg/ml on admission to 1843 (118-10815) pg/ml and 1236 (332-11100) pg/ml on days 3 and 5, respectively (p > 0,05). The plasma concentration of NT-proBNP on admission in patients with mild ARDS was 325 (107-4059) pg/ml vs. 17541 (3797-40795) pg/ml in the group of patients with moderate and severe ARDS (p = 0,028). On admission, NT-proBNP correlated with GEDVI (rho = 0,615; p = 0,025), which was 701 (652-910) ml/m^2^. We did not find correlation of plasma NT-proBNP with CI and EVLW.

## Conclusions

In ARDS patients, we observed increased plasma concentrations of NT-proBNP on admission with a trend for decrease over time. In moderate and severe ARDS, NT-proBNP concentrations are higher than in mild ARDS. The plasma concentrations of NT-proBNP correlate with GEDVI; however we observed no relationship of NT-proBNP with CI and EVLW. Thus, NT-proBNP has a limited value to diagnose pulmonary edema in ARDS.

